# UNIVERSITY-CITY PARTNERSHIP TO RECRUIT DIVERSE, LOW-INCOME SENIORS TO ALLEVIATE HEALTH DISPARITIES

**DOI:** 10.1093/geroni/igae098.3918

**Published:** 2024-12-31

**Authors:** Carla Leinbach, Abigail Tice, Janet Lopez

**Affiliations:** University of Central Florida, Orlando, Florida, United States; University of Central Florida, Orlando, Florida, United States; University of Central Florida, Orlando, Florida, United States

## Abstract

Recruitment challenges are the top cited reason that research studies miss their goals. Recruiting under-represented groups including people of color, those with cognitive or physical challenges, socio-economically marginalized persons, and older persons exacerbates recruitment challenges. We aimed to explore strategies to recruit diverse, low-income, older participants for our NIH-funded clinical trial study and compared recruitment numbers between pre-post recruitment implementations. We collected data from May to August 2024 from Orlando, Florida. Pre-implementation, locations had fewer than 10 participants per site and required 8 to 12 weeks to organize. Our findings indicated four recruitment strategies 1) creating university-city partnerships resulted in 17 new senior programs to recruit from; 2) recruiting untapped places resulted in an uptick in new participants; 3) Multi-lingual marketing increased enrollment inquiries, and 4) Efficiently captured inquiries increased enrollment. Pre-implementation, the study struggled to identify locations with sufficient senior population density, identified limited physical spaces to conduct assessments, struggled to obtain permission to recruit on-site, and faced market rental rates for use of space. Enrollment rates were low due to the facilities’ leadership and potential participants viewed researchers as “vendors”, not trusted partners. Post-implementation, cohorts ranged from 25 – 40+ seniors and required approximately 2 weeks to organize. This partnership expanded the reach of recruitment into historically difficult to access populations and contributed to a net regain of committed completion timelines. Strategic utilization of local city and county government for participant recruitment and facility access can aid research projects in reaching their goals and diversifying the participant pool.

